# Green Space and Internalizing or Externalizing Symptoms Among Children

**DOI:** 10.1001/jamanetworkopen.2024.5742

**Published:** 2024-04-10

**Authors:** Nissa Towe-Goodman, Kristen L. McArthur, Michael Willoughby, Margaret M. Swingler, Cara Wychgram, Allan C. Just, Itai Kloog, Deborah H. Bennett, Daniel Berry, Marnie F. Hazlehurst, Peter James, Marcia Pescador Jimenez, Jin-Shei Lai, Leslie D. Leve, Lisa Gatzke-Kopp, Julie B. Schweitzer, Traci A. Bekelman, Catrina Calub, Susan Carnell, Sean Deoni, Viren D’Sa, Carrie Kelly, Daphne Koinis-Mitchell, Michael Petriello, Gita Thapaliya, Rosalind J. Wright, Xueying Zhang, Amii M. Kress

**Affiliations:** 1 Frank Porter Graham Child Development Institute, University of North Carolina at Chapel Hill, Chapel Hill; 2 Department of Epidemiology, Bloomberg School of Public Health, Johns Hopkins University, Baltimore, Maryland; 3 Education and Workforce Development, RTI International, Research Triangle Park, North Carolina; 4 Department of Epidemiology, Institute at Brown for Environment and Society, Brown University, Providence, Rhode Island; 5 Department of Environmental Medicine and Public Health, Icahn School of Medicine at Mount Sinai, New York, New York; 6 Department of Public Health Sciences, University of California, Davis, Sacramento; 7 Institute of Child Development, University of Minnesota, Minneapolis; 8 Department of Environmental & Occupational Health Sciences, School of Public Health, University of Washington, Seattle; 9 Department of Population Medicine, Harvard Medical School and Harvard Pilgrim Health, Boston, Massachusetts; 10 Department of Epidemiology, Boston University School of Public Health, Boston, Massachusetts; 11 Department of Medical Social Sciences, Northwestern University, Chicago, Illinois; 12 Prevention Science Institute, University of Oregon, Eugene; 13 Department of Human Development and Family Studies, The Pennsylvania State University, University Park; 14 Lifecourse Epidemiology of Adiposity and Diabetes (LEAD) Center, University of Colorado Anschutz Medical Campus, Aurora; 15 Maternal, Newborn, and Child Health Discovery & Tools, Bill & Melinda Gates Foundation, Seattle, Washington; 16 Department of Pediatrics, Rhode Island Hospital, Warren Alpert Medical School of Brown University, Providence, Rhode Island; 17 Institute of Environmental Health Sciences, Department of Pharmacology, Wayne State University, Detroit, Michigan; 18 Department of Pediatrics, Icahn School of Medicine at Mount Sinai, New York, New York

## Abstract

**Importance:**

Evidence suggests that living near green space supports mental health, but studies examining the association of green space with early mental health symptoms among children are rare.

**Objective:**

To evaluate the association between residential green space and early internalizing (eg, anxiety and depression) and externalizing (eg, aggression and rule-breaking) symptoms.

**Design, Setting, and Participants:**

Data for this cohort study were drawn from the Environmental Influences on Child Health Outcomes cohort; analysis was conducted from July to October 2023. Children born between 2007 and 2013 with outcome data in early (aged 2-5 years) and/or middle (aged 6-11 years) childhood who resided in 41 states across the US, drawing from clinic, hospital, and community-based cohorts, were included. Cohort sites were eligible if they recruited general population participants and if at least 30 children had outcome and residential address data to measure green space exposure. Nine cohorts with 13 sites met these criteria. Children diagnosed with autism or developmental delay were excluded, and 1 child per family was included.

**Exposures:**

Green space exposure was measured using a biannual (ie, summer and winter) Normalized Difference Vegetation Index, a satellite image–based indicator of vegetation density assigned to monthly residential history from birth to outcome assessment.

**Main Outcome and Measures:**

Child internalizing and externalizing symptoms were assessed using the Child Behavior Checklist for Ages 1½ to 5 or 6 to 18. The association between green space and internalizing and externalizing symptoms was modeled with multivariable linear regression using generalized estimating equations, adjusting for birthing parent educational level, age at delivery, child sex, prematurity, and neighborhood socioeconomic vulnerability. Models were estimated separately for early and middle childhood samples.

**Results:**

Among 2103 children included, 1061 (50.5%) were male; 606 (29.1%) identified as Black, 1094 (52.5%) as White, 248 (11.9%) as multiple races, and 137 (6.6%) as other races. Outcomes were assessed at mean (SD) ages of 4.2 (0.6) years in 1469 children aged 2 to 5 years and 7.8 (1.6) years in 1173 children aged 6 to 11 years. Greater green space exposure was associated with fewer early childhood internalizing symptoms in fully adjusted models (*b* = −1.29; 95% CI, −1.62 to −0.97). No associations were observed between residential green space and internalizing or externalizing symptoms in middle childhood.

**Conclusions and Relevance:**

In this study of residential green space and children’s mental health, the association of green space with fewer internalizing symptoms was observed only in early childhood, suggesting a sensitive period for nature exposure. Policies protecting and promoting access to green space may help alleviate early mental health risk.

## Key Points

**Question:**

Is exposure to green space associated with internalizing (eg, anxiety and depression) and externalizing (eg, aggression and rule-breaking) symptoms among children?

**Findings:**

In this cohort study of 2103 children in 41 states across the US, greater residential green space exposure was associated with fewer internalizing symptoms in early childhood but not in middle childhood.

**Meaning:**

These findings suggest that green space initiatives may help reduce the risk of early anxiety and depressive symptoms in children across the US.

## Teaser

This cohort study of children born between 2007 and 2013 examines associations between residential green space exposure and internalizing and externalizing symptoms in early and middle childhood.

## Introduction

The mental health of children in the US is a national emergency.^1^ Up to 40% of children will meet the criteria for a mental disorder by adulthood,^2^ an epidemic that has only accelerated in recent years.^3^ Impaired mental health is even more common below diagnostic thresholds,^4^ with notable increases in internalizing (eg, anxiety and depression) and externalizing (eg, aggression and rule-breaking) symptoms.^5^ Identifying environmental factors that buffer children from internalizing and externalizing symptoms offers the potential for modifiable pathways to offset risk.

Exposure to nature may be one such pathway. Forests, parks, backyards, and other green spaces offer children opportunities to restore emotional and physiologic resources; build regulatory capacities through risk-taking, physical activity, and play; and reduce harm from environmental stressors, such as heat or air pollution.^6^ Experimental research notes short-term benefits of green space on improved mood and physiologic and perceived indicators of stress.^7,8^ Longitudinal evidence indicates links between green space and mental health^7,9,10^; adolescents and adults raised in low levels of green space have up to a 55% greater risk for mental disorders than those raised in high levels of green space.^11^ However, studies examining early emerging internalizing and externalizing symptoms are rare. This is a notable gap: plasticity in emotional, physiologic, and behavioral regulation peaks in early childhood and is shaped by environmental experiences,^12,13^ and early emerging symptoms have prolonged effects on functioning.^14,15^ Additionally, research to date is predominantly cross-sectional, focused within single or small groups of cities, and typically fails to account for neighborhood socioeconomic vulnerability, a co-occurring environmental risk.^10,16,17^ Evidence suggests that green space and socioeconomic vulnerability have independent links with health outcomes^18^ despite disparities in availability^17^; the protective benefits of green space may be greater in low-income areas, offsetting social and environmental stressors.^19^ No studies to date have examined the associations between residential green space exposure from birth, neighborhood socioeconomic vulnerability, and early and middle childhood internalizing and externalizing symptoms across the US. Such research could inform feasible interventions and policy changes to promote the development of children in the US, reducing the rising burden of mental illness.

To address this gap, the current study leveraged data from the Environmental Influences on Child Health Outcomes (ECHO) cohort, a National Institutes of Health–funded consortium of socioeconomically and geographically diverse cohort sites across the US studying the environmental factors contributing to child health, to examine associations between residential green space exposure and internalizing and externalizing symptoms in early (ages 2-5 years) and middle (ages 6-11 years) childhood. A secondary aim was to examine whether neighborhood socioeconomic vulnerability or child sex modified observed associations between green space and internalizing or externalizing symptoms.

## Methods

### Study Population

The ECHO cohort comprises 69 ongoing cohorts across the US.^20^ Children born between 2007 and 2013 who recruited to a general cohort (ie, not selected on medical risk or adoption status) were eligible for the current analysis if they had 1 birthing parent report of internalizing and/or externalizing symptoms via the Child Behavior Checklist for Ages 1½ to 5 years or 6 to 18 years (CBCL 1½-5 or 6-18)^21,22^ before March 15, 2020 (1 child per family), and a high-quality-match geocoded residential address (point or specific street address, >85% of geocoded addresses) for more than 75% of months from birth to outcome assessment. Children diagnosed with autism spectrum disorder or developmental delay were excluded (n = 240); associations between green space and mental health may be more complex within these populations.^23^ Cohort enrollment sites with fewer than 30 eligible participants were excluded (9 sites, n = 90; eFigure 1 in Supplement 1). Nine cohorts met these criteria, representing 13 clinic, hospital, and community sites and 2103 children (eTable 1 in Supplement 1); children resided in 199 counties across 41 states (Figure, A). The early childhood (ages 2-5 years) sample included 1469 children from 6 cohorts (7 sites) with 1 measure of internalizing or externalizing symptoms. The middle childhood (ages 6-11 years) sample included 1173 children from 7 cohorts (10 sites); 539 participants (25.6%) who had CBCL data in both early and middle childhood were included in both sets of analyses, almost exclusively from a single cohort (523 [97.0%]). Child race and ethnicity were included as descriptors; these data were provided by the caregiver and harmonized across samples as ethnicity being Hispanic or non-Hispanic and primary race being Black, White, more than 1 race, or other (Alaska Native, American Indian, Asian, Native Hawaiian or other Pacific Islander, or other). The study protocol was reviewed and approved by the local (or single ECHO) institutional review board. Written informed parental consent or permission was obtained along with child assent as appropriate for ECHO-wide and cohort participation. This manuscript follows the Strengthening the Reporting of Observational Studies in Epidemiology (STROBE) reporting guideline.

**Figure.**
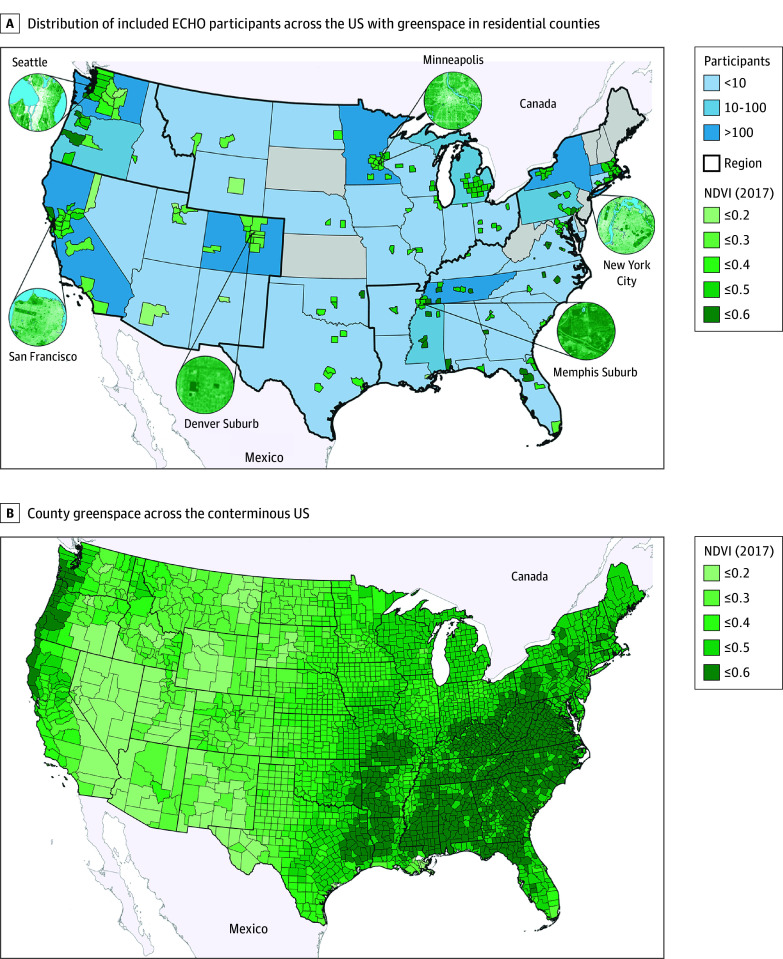
Maps of Included Environmental Influences on Child Health Outcomes (ECHO) Participants and County-Level Summer and Winter Green Space Across the Conterminous US Mean summer and winter Normalized Difference Vegetation Index (NDVI) values in 2017 for residential counties of ECHO participants and counties across the conterminous US.

### Measures

#### Green Space

A monthly residential address history file was generated for each child, accounting for moves from birth to the outcome assessment. Addresses were geocoded using ArcGIS Streetmap Premium, version 3.0 (Esri), generating latitude and longitude for addresses to link with green space data. Monthly green space was estimated using the summer and winter Normalized Difference Vegetation Index (NDVI), a satellite image–based indicator of relative live vegetation density widely used in epidemiologic research.^24,25^ The NDVI values range from −1 to 1; higher values represent dense vegetation (eg, forests), values close to 0 represent areas without live vegetation, and negative values represent water bodies. Google Earth Engine was used to generate cloud-free ultra-high-resolution (30 m) NDVI values from Landsat satellite data across the conterminous US using XIS (XGBoost-IDW Synthesis^26^); select ultra-high-resolution images within sample counties (insets) and national county-level summer and winter average NDVI values are presented in the Figure, A and B, respectively. Residential greenness was processed using Python software, version 3.9.11 (Python Software Foundation) and ArcGIS, with average NDVI values estimated in buffers of 270, 510, and 1230 m around home addresses to evaluate the immediate residential area, nearby neighborhood, and extended walkable areas. Consistent with prior research,^17^ negative NDVI values were set to 0 (<5 children), and raw NDVI values were rescaled by multiplying values greater than 0 by 10 for modeling and interpretability. The NDVI values were assigned to monthly address history to create time-weighted average green space from birth to outcome accounting for residential mobility; NDVI measures (summer) were highly correlated over time within children (intraclass correlation coefficients of 0.84 and 0.81 in early and middle childhood samples, respectively). The NDVI values in buffers of 270 m are reported in the main analyses.

#### Child Internalizing and Externalizing Symptoms

Preschool (early) or school-aged (middle) CBCL 1½-5 or 6-18 assessed internalizing (ie, anxiety, depression, withdrawal, and somatic concerns) and externalizing (ie, rule-breaking and aggressive behavior) symptoms,^21,22^ which include 100 and 119 items, respectively. The CBCL 1½-5 or 6-18 is extensively used as a reliable and valid assessment of internalizing and externalizing symptoms^27^ associated with concurrent and adult clinical health^28,29^; given significant discrepancies across parental reports on the CBCL,^30^ we used birthing parent ratings of symptoms observed in the past 6 months, with item responses ranging from 0 (not true) to 2 (very true or often true). Nationally normed T scores based on age and sex were used in the current analysis.

#### Confounders

Neighborhood- and individual-level confounders were identified a priori based on theory, research, and data availability. Neighborhood socioeconomic status (SES) vulnerability was assessed using the SES theme of the Social Vulnerability Index,^31^ which includes rankings of census tracts based on census-reported levels of poverty, unemployment, housing cost burden, no high school diploma, and no health insurance. Scores reflect the proportion of national tracts less vulnerable than the residential tract (higher scores reflect greater vulnerability based on low SES). Social Vulnerability Index values averaged for the exposure period were categorized into tertiles established by national data representing low, medium, and high vulnerability based on US norms.^32^ At the individual level, we adjusted for birthing parent age at delivery (continuous) and educational level (highest attained: less than high school, high school or equivalent, or more than high school) as well as child sex assigned at birth and preterm status (<37 vs ≥37 weeks’ gestation).

### Statistical Analysis

Analyses were conducted from July to October 2023 using Stata software, version 17 (StataCorp LLC)^33^ and ArcGIS Pro, version 3.0.3 (Esri).^34^ We examined outcome distributions to ensure normality and the functional form of the association between NDVI and outcomes with scatterplots and Lowess lines. Because most cohorts had CBCL assessments in either early or middle childhood (but not both), all analyses were run separately for the early and middle childhood samples. Because ECHO data are clustered by design, we used multivariable linear regression with generalized estimating equation (exchangeable correlation structure and robust variance) estimates to account for clustering of participants within cohort enrollment sites. We first examined unadjusted models of associations between NDVI (per 0.1 increments) and internalizing or externalizing symptoms, then adjusted for individual-level confounders, and finally adjusted for individual- and area-level SES vulnerability. Because more than 99% of the sample had complete covariate data, we used complete case analysis for adjusted models. To aid in interpretation, we used model estimates to calculate the difference in estimated CBCL for individuals residing at relatively high green space compared with low (90th vs 10th percentile NDVI for sample).

We conducted 2 sets of a priori exploratory analyses examining effect measure modification by child sex assigned at birth and by average neighborhood SES vulnerability; interaction terms were added in the fully adjusted models. We considered an interaction term 2-sided *P* = .10 as the threshold for effect measure modification.

We evaluated the robustness of the results through sensitivity analyses. We examined associations between NDVI and internalizing and externalizing symptoms in buffers of 510 and 1230 m to evaluate the sensitivity of associations to the size of the buffer used. Next, the relative contribution to the estimates from a single cohort enrollment site were evaluated using the leave-one-out approach.

## Results

Among 2103 children included (1469 in the early childhood sample, 1173 in the middle childhood sample, and 539 children in both samples), 1061 (50.5%) were male and 1042 (49.5%) female; 203 (9.7%) were preterm and 1900 (90.3%) were not; 606 (29.1%) were Black, 1094 (52.5%) were White, 248 (11.9%) were of more than 1 race, and 137 (6.6%) were of other races (Alaska Native, American Indian, Asian, Native Hawaiian or other Pacific Islander, or caregiver-reported “other” race); and 1761 (84.0%) were non-Hispanic and 336 (16.0%) Hispanic. Most birthing parents (1795 [85.4%]) had more than a high school education, with a mean (SD) age of 29.0 (6.0) years at delivery. Descriptive statistics for the early and middle childhood samples are presented in Table 1 (eTables 2-3 in Supplement 1). Symptoms were reported at a mean (SD) age of 4.2 (0.6) years for the early childhood sample and 7.8 (1.6) years for the middle childhood sample. Most children moved at least once between birth and outcome assessment (855 [58.2%] and 799 [68.1%] in the early and middle childhood samples, respectively). There were not notable differences in demographics between the early and middle childhood analytic samples.

**Table 1.** Child, Parental, and Residential Characteristics by Early and Middle Childhood Study Populations^a^

Characteristic	Early childhood (n = 1469)	Middle childhood (n = 1173)	Total (N = 2103)
Child characteristics			
Age at CBCL 1½-5 or 6-18 assessment, mean (SD), y	4.2 (0.6)	7.8 (1.6)	5.0 (1.5)
CBCL 1½-5 or 6-18 internalizing symptoms T score, median (IQR)	45 (37-53)	48 (41-54)	45 (39-54)
CBCL 1½-5 or 6-18 externalizing symptoms T score, median (IQR)	44 (39-51)	48 (41-56)	46 (39-53)
Sex assigned at birth			
Male	745 (50.7)	581 (49.5)	1061 (50.5)
Female	724 (49.3)	592 (50.5)	1042 (49.5)
Child race^b^			
Black	528 (36.3)	416 (35.6)	606 (29.1)
White	697 (47.9)	558 (47.8)	1094 (52.5)
>1 Race	139 (9.5)	143 (12.3)	248 (11.9)
Other^c^	92 (6.3)	50 (4.3)	137 (6.6)
Child ethnicity^b^			
Non-Hispanic	1215 (83.0)	1068 (91.1)	1761 (84.0)
Hispanic	249 (17.0)	104 (8.9)	336 (16.0)
Preterm (<37 completed weeks)			
No	1354 (92.2)	1038 (88.5)	1900 (90.3)
Yes	115 (7.8)	135 (11.5)	203 (9.7)
Birthing parent characteristics			
Age at delivery, mean (SD), y	29.0 (6)	29.0 (6.0)	29.0 (6.0)
Educational attainment			
Less than high school	59 (4.0)	27 (2.3)	77 (3.7)
High school, GED, or equivalent	181 (12.3)	132 (11.3)	229 (10.9)
Some college or more	1228 (83.7)	1013 (86.4)	1795 (85.4)
Residential characteristics			
Average neighborhood SES (SVI)			
High	536 (36.5)	497 (42.4)	885 (42.1)
Medium	339 (23.1)	266 (22.7)	489 (23.3)
Low	594 (40.4)	410 (35.0)	729 (34.7)
Region			
West	413 (28.1)	194 (16.5)	606 (28.8)
Northwest	84 (5.7)	184 (15.7)	268 (12.7)
Midwest or Central	87 (5.9)	117 (10.0)	204 (9.7)
East	885 (60.2)	678 (57.8)	1025 (48.7)
NDVI at 270 m from birth to outcome, mean (SD)	0.32 (0.08)	0.35 (0.08)	0.32 (0.09)
Movers			
No (1 residential location)	614 (41.8)	374 (31.9)	988 (37.4)
Yes (>1 residential location)	855 (58.2)	799 (68.1)	1654 (62.6)

Abbreviations: CBCL 1½-5 or 6-18, Child Behavior Checklist for Ages 1½ to 5 or 6 to 18; GED, General Educational Development; NDVI, Normalized Difference Vegetation Index; SES, socioeconomic status; SVI, Social Vulnerability Index.

^a^ Data are presented as number (percentage) of participants unless otherwise indicated. Covariate data are complete or are missing at less than 1%; 539 participants are included in both the early and middle childhood samples.

^b^ Child race and ethnicity were identified through caregiver report.

^c^ Other includes Alaska Native, American Indian, Asian, Native Hawaiian or other Pacific Islander, or caregiver-reported “other.”

Most children were recruited from cohort sites in the Eastern US (885 [60.2%] and 678 [57.8%] for early and middle childhood samples, respectively). More than one-third (885 [42.1%]) of the children resided in neighborhoods classified as high SES vulnerability. Green space was negatively correlated with internalizing and externalizing symptoms in early childhood (*r* = −0.12 and *P* < .001 and *r* = −0.08 and *P* = .002, respectively) but not in middle childhood (*r* = 0.01 and *P* = .78 and *r* = 0.02 and *P* = .59, respectively).

Unadjusted and adjusted models are presented in Table 2. A 0.1-unit higher NDVI at 270 m was associated with 1.28-unit lower internalizing (*b* = −1.28; 95% CI, −1.62 to −0.95) and 0.77-unit lower externalizing (*b* = −0.77; 95% CI, −11.53 to −0.02) T scores in early childhood (Table 2), adjusting for child sex assigned at birth, preterm status, birthing parent educational level, and age at delivery. The association held for internalizing symptoms after adjustment for neighborhood SES vulnerability (*b* = −1.29; 95% CI, −1.62 to −0.97) but was attenuated for externalizing symptoms (*b* = −0.66; 95% CI, −1.38 to 0.06); estimates for covariates in fully adjusted models are presented in eTable 4 in Supplement 1. In fully adjusted models, children residing at relatively high green space (90th percentile NDVI = 0.42) had mean CBCL T scores 2.62 points lower for internalizing and 1.34 points lower for externalizing symptoms compared with children residing in lower levels (10th NDVI = 0.22); example counties with average NDVI values at the 90th and 10th percentiles are shown in eFigure 2 in Supplement 1. In middle childhood, analyses revealed no associations between green space and internalizing or externalizing scores, which did not change after adjustment for individual- and/or area-level factors.

**Table 2.** Adjusted Association Between Residential Green Space and Children’s Internalizing and Externalizing Symptoms (CBCL 1½-5 or 6-18 T Score)^a^

NDVI	*b* (95% CI)					
	Internalizing symptoms			Externalizing symptoms		
	Unadjusted	Individual-level covariates	Individual-level covariates and average SVI	Unadjusted	Individual-level covariates	Individual-level covariates and average SVI
**Early childhood (aged 2-5 y)**						
No.	1469	1463	1463	1469	1463	1463
Average NDVI within 270 m	−1.59 (−2.28 to −0.87)^b^	−1.28 (−1.62 to −0.95)^b^	−1.29 (−1.62 to −0.97)^b^	−1.16 (−2.10 to −0.22)^b^	−0.77 (−11.53 to −0.02)^b^	−0.66 (−1.38 to 0.06)
Average NDVI within 510 m^c^	−1.59 (−2.40 to −0.79)^b^	−1.26 (−0.69 to −0.84)^b^	−1.27 (−1.67 to −0.87)^b^	−1.25 (−2.19 to −0.30)^b^	−0.83 (−1.62 to −0.05)^b^	−0.69 (−1.47 to 0.08)
Average NDVI within 1230 m^c^	−1.38 (−2.29 to −0.47)^b^	−1.10 (−1.59 to −0.60)^b^	−1.10 (−1.58 to −0.62)^b^	−1.05 (−2.00 to −0.10)^b^	−0.62 (−1.40 to 0.17)	−0.48 (−1.22 to 0.27)
**Middle childhood (aged 6-11 y)**						
No.	1172	1170	1170	1171	1169	1169
Average NDVI within 270 m	0.16 (−0.80 to 1.12)	0.11 (−0.81 to 1.03)	0.11 (−0.70 to 0.92)	0.18 (−0.34 to 0.71)	0.19 (−0.44 to 0.82)	0.20 (−0.51 to 0.91)
Average NDVI within 510 m^c^	0.22 (−0.82 to 1.26)	0.15 (−0.85 to 1.16)	0.12 (−0.79 to 1.02)	0.20 (−0.31 to 0.70)	0.22 (−0.40 to 0.84)	0.25 (−0.45 to 0.96)
Average NDVI within 1230 m^c^	0.26 (−0.69 to 1.21)	0.16 (−0.79 to 1.11)	0.09 (−0.81 to 0.99)	0.07 (−0.55 to 0.69)	0.09 (−0.63 to 0.80)	0.13 (−0.64 to 0.90)

Abbreviations: CBCL 1½-5 or 6-18, Child Behavior Checklist for Ages 1½ to 5 or 6 to 18; NDVI, Normalized Difference Vegetation Index; SVI, Social Vulnerability Index.

^a^ Coefficients (per 0.1 higher NDVI) from generalized estimating equation linear regression of the association between average green space exposure from birth and internalizing and externalizing symptoms. Models are (1) unadjusted and then adjusted for (2) individual-level confounders (birthing parent’s educational level, age at delivery, child sex, and preterm birth status) and (3) individual confounders and tract-level Social Vulnerability Index from birth. High social vulnerability corresponds with low socioeconomic status.

^b^ Statistically significant based on *P* < .05.

^c^ Sensitivity analysis of different buffer sizes for comparability with previous literature.

No evidence of effect measure modification by child sex emerged for internalizing or externalizing symptoms. Associations were also similar across high vs low neighborhood SES vulnerability. Although there was a significant interaction between moderate SES vulnerability and green space compared with low SES vulnerability for middle childhood externalizing symptoms, we were unable to run analyses stratified by neighborhood SES vulnerability due to sparseness across cells.

Sensitivity analyses were conducted to investigate the robustness of associations. In early childhood, a similar pattern emerged at 510-m and 1230-m buffers, although the magnitude of associations was attenuated at 1230 m. Sensitivity analyses for middle childhood similarly found no association of green space with internalizing or externalizing symptoms at larger buffers. To assess whether associations were robust and not driven by any single site, fully adjusted models were reestimated, leaving 1 enrollment site out at a time. Forest plots of estimates for early and middle childhood internalizing and externalizing symptoms are summarized in eFigure 3 in Supplement 1.

## Discussion

To our knowledge, this is the first study to examine the association of green space exposure on early internalizing and externalizing symptoms in children across the US. Our results suggest that higher levels of residential green space are associated with fewer internalizing symptoms in early childhood (aged 2-5 years) before and after adjusting for child sex, prematurity, birthing parent educational level and age at delivery, and neighborhood socioeconomic vulnerability. Although green space was also associated with externalizing problems in early childhood, this association was attenuated after accounting for neighborhood socioeconomic vulnerability. We did not find evidence of associations between residential green space and internalizing or externalizing symptoms in middle childhood (aged 6-11 years). Leveraging data from multiple diverse cohorts with substantial regional, demographic, and economic diversity, these findings extend our understanding of nature as a protective factor for mental health risk.

Our finding that green space is associated with fewer early childhood internalizing symptoms is consistent with evidence of the benefits of nature on children’s functioning^10^ and work, suggesting that early childhood may be a sensitive period for green space exposure.^32^ In fully adjusted models, residing in relatively low vs high levels of green space was associated with 2.62-point higher internalizing T scores; this association is greater in magnitude than risks such as exceeding the recommended threshold of screentime (>2 hours per day) or the benefit of more than 2 hours per week of organized physical activity on early internalizing scores.^35^ Rapid neural maturation and foundational regulatory skill development make early childhood a sensitive window for environmental influences. With the home as a central context for early development, nearby nature may offer unique opportunities for positive emotions, stress reduction, and the restoration of emotional resources through evolutionarily determined pathways; areas directly around the residence may be particularly salient for young children given the restorative influence of green space within visible distances around the home.^6^ Additionally, green space may reduce the impact of environmental stressors such as heat or air pollution, increase exposure to diverse microbiomes, and offer opportunities for play and physical activity,^36,37^ providing multiple pathways to offset mental health risk. The protection and extension of natural environments to young children may offer widespread benefits.

Notably, associations between green space and early childhood internalizing symptoms were stronger than for externalizing symptoms. This discrepancy was more pronounced after accounting for neighborhood socioeconomic vulnerability; because there are notable socioeconomic disparities in green space^17^ and neighborhood poverty has stronger links with externalizing than internalizing symptoms,^38^ this finding is not entirely surprising. Nationally representative studies from Australia and Europe have also reported stronger associations between green space and children’s internalizing symptoms,^39^ depression diagnoses,^40^ and internalizing disorders into adulthood,^11^ although findings are mixed within adolescence.^41^ Although externalizing symptoms were not examined, Bezold et al^42^ found links between higher greenness and lower risk for depressive symptoms in more than 11 000 adolescents and adults across the US. In one of the only longitudinal studies focused on preschool, green space similarly supported resilience for boys’ emotional symptoms.^43^ Because early internalizing symptoms may have a prolonged and severe developmental course,^15^ the protective role of green space within this period may have long-term implications.

The lack of associations between residential green space and middle childhood internalizing or externalizing symptoms is counter to the broader body of green space research to date. However, middle childhood mental health research is lagging in general^44^; research from school age through adolescence suggests that the magnitude of green space associations vary across development.^45^ The transition to school represents a notable change in the environment for children, and school green space exposure may be an important influence.^46^ For example, Liao et al^47^ found that the combination of concurrent school and residential greenness was linked with kindergarten mental health symptoms, but residential green space alone was not. Time-use data may offer more accurate exposure estimates, with free-play at home decreasing across childhood^48^ and participation in extracurricular activities at home or elsewhere varying according to parental values and SES.^49^ Additionally, inclusion of self-report or teacher report could improve outcome assessment in middle childhood, particularly for internalizing symptoms,^50^ and the middle childhood sample had differences in both regional characteristics and socioeconomic vulnerability. Improving the accuracy of exposure and outcome measurement across diverse samples is an important area for investigation.

With data pooled across all sites, green space near the home had similar and independent associations with early internalizing symptoms across child sex and across the most vs the least socioeconomically disadvantaged neighborhoods. Consistent protective linkages with green space are in line with prior work indicating unique associations of SES and green space on child outcomes^11^; the potential for universal benefits of nearby natural areas on early childhood mental health is promising.

### Limitations

Confounding factors may have influenced our results, such as exposure to environmental hazards, parenting quality, or neighborhood cohesion or violence. Although neighborhood SES vulnerability and individual characteristics were included in our models, choice of residence may be driven by historical social class, residential segregation, or additional factors not included. The NDVI does not indicate the quality, accessibility, or use of nearby nature, and we did not assess childcare or school green space. Similarly, characteristics of green space that may be important for mental health were not assessed, such as woodland composition, water, or biodiversity. The sample predominantly resided in the East and metropolitan areas; these findings may not generalize across rural or other areas. Associations between green space and internalizing symptoms were modest; social or genetic factors may have stronger implications for early symptoms. Although our assessment of average green space exposure from birth to the outcome assessment incorporated shifts in NDVI values due to residential moves or urbanization over time, longitudinal research is needed to examine the dynamic changes in green space and the development of internalizing and externalizing symptoms across childhood.

## Conclusions

Residential green space exposure was associated with fewer early childhood internalizing symptoms within the national ECHO-wide cohort, even after accounting for neighborhood socioeconomic vulnerability. These findings suggest that green initiatives (eg, parks, urban forest programs, and protected natural areas) have the potential to reduce risk for early anxiety and depressive symptoms in children across the US. At a time of crisis in children’s mental health and dwindling natural environments, policies that protect and promote green space could have widespread benefits for children, society, and the environment.

## Notes

Supplement 1. **eTable 1.** Included ECHO Cohort Descriptions **eTable 2.** Distribution of Child, Parental, and Residential Characteristics for Early Childhood Study Population by Quartiles of NDVI (n = 1469) **eTable 3.** Distribution of Child, Parental, and Residential Characteristics for Middle Childhood Study Population by Quartiles of NDVI (n = 1173) **eTable 4.** Fully Adjusted Associations Between Residential Green Space, Individual and Area-Level Confounders, and Children’s Internalizing and Externalizing Symptoms (CBCL 1½-5/6-18 t-Score) **eFigure 1.** Flowchart for Final Analytic Sample of 2103 Children Included in the Analysis of Residential Green Space and Internalizing and Externalizing Symptoms **eFigure 2.** Example Counties With Average NDVI Values at Relatively High Levels of Greenspace (at the 90th Percentile of NDVI) for the Early Childhood Sample Compared to Relatively Low Levels (the 10th Percentile) **eFigure 3.** Leave-One-Out (Cohort Enrollment Site) Coefficient Plots for Association Between Green Space (270m) Internalizing and Externalizing Symptoms for Early and Middle Childhood Samples

Supplement 2.
Nonauthor Collaborators

Supplement 3.
Data Sharing Statement
