# Supplementary material for: Green Space and Internalizing or Externalizing Symptoms Among Children
Source: JAMA Netw Open. 2024 Apr 10;7(4):e245742. doi: 10.1001/jamanetworkopen.2024.5742 (PMC11007572; doi:10.1001/jamanetworkopen.2024.5742)
Supplement: Supplement 1. — eTable 1. Included ECHO Cohort Descriptions eTable 2. Distribution of Child, Parental, and Residential Characteristics for Early Childhood Study Population by Quartiles of NDVI (n = 1469) eTable 3. Distribution of Child, Parental, and Residential Characteristics for Middle Childhood Study Population by Quartiles of NDVI (n = 1173) eTable 4. Fully Adjusted Associations Between Residential Green Space, Individual and Area-Level Confounders, and Children’s Internalizing and Externalizing Symptoms (CBCL 1½-5/6-18 t-Score) eFigure 1. Flowchart for Final Analytic Sample of 2103 Children Included in the Analysis of Residential Green Space and Internalizing and Externalizing Symptoms eFigure 2. Example Counties With Average NDVI Values at Relatively High Levels of Greenspace (at the 90th Percentile of NDVI) for the Early Childhood Sample Compared to Relatively Low Levels (the 10th Percentile) eFigure 3. Leave-One-Out (Cohort Enrollment Site) Coefficient Plots for Association Between Green Space (270m) Internalizing and Externalizing Symptoms for Early and Middle Childhood Samples [file jamanetwopen-e245742-s001.pdf]

Supplementary Online Content

Towe-Goodman N, McArthur KL, Willoughby M, et al. Greenspace and internalizing and externalizing symptoms among children. *JAMA Netw Open*. 2024;7(4):e245742. doi:10.1001/jamanetworkopen.2024.5742

eTable 1. Included ECHO Cohort Descriptions

eTable 2. Distribution of Child, Parental, and Residential Characteristics for Early Childhood Study Population by Quartiles of NDVI (n = 1,469)

eTable 3. Distribution of Child, Parental, and Residential Characteristics for Middle Childhood Study Population by Quartiles of NDVI (n = 1,173)

eTable 4. Fully Adjusted Associations Between Residential Greenspace, Individual and Area-Level Confounders, and Children's Internalizing and Externalizing Symptoms (CBCL 1½-5/6-18 t-Score)

eFigure 1. Flowchart for Final Analytic Sample of 2,103 Children Included in the Analysis of Residential Greenspace and Internalizing and Externalizing Symptoms

eFigure 2. Example Counties With Average NDVI Values at Relatively High Levels of Greenspace (at the 90th Percentile of NDVI) for the Early Childhood Sample Compared to Relatively Low Levels (the 10th Percentile)

eFigure 3. Leave-One-Out (Cohort Enrollment Site) Coefficient Plots for Association Between Greenspace (270m) Internalizing and Externalizing Symptoms for Early and Middle Childhood Samples

This supplementary material has been provided by the authors to give readers additional information about their work.

© 2024 Towe-Goodman N et al. *JAMA Network Open.*

1 eTable 1. Included ECHO Cohort Descriptions.

Cohort	**Early Childhood** n = 1,469; 6 cohorts, 7 enrollment sites	**Middle Childhood** n = 1,173; 7 cohorts, 10 enrollment sites	Sample Description	Study Aims
Healthy Start	**N=413**	**N < 50***	Colorado community sample of mother-child dyads recruited in infancy from obstetrics clinics at a university hospital and by word of mouth, as well as medical university employees.	To understand the contribution of metabolic and behavioral factors during pregnancy to the development of childhood obesity, insulin resistance, and inflammatory markers.
Brown University Assessment of Myelination and Behavior Across Maturation (BAMBAM)	**N=72**	**N< 50***	Children recruited between 3 months to 12 years of age from the general population recruited in pediatrician offices and via online webpages, radio advertisements, and flyers in Providence, RI.	To examine typical brain development, including how brain growth is altered by specific pre- and post-natal environmental or genetic factors; how patterns of brain growth are associated with, and predictive of, emerging cognitive and behavioral abilities; and how these brain-behavior relationships are influenced by modifiable factors experienced throughout childhood.
Revisiting Childhood Autism Risks from Genes and the Environment Study (ReCHARGE)		**N< 50***	Children ages 2-5 years with a diagnosis of autism spectrum disorder (ASD) or another developmental disorder identified from the California Department of Developmental Services list of individuals receiving services for ASD or other neurobehavioral delays, from regional service coordination centers, or referrals from clinics or self-referral; Control children were sampled from birth files matched on demographics to the ASD sample. Only control children from the general	To identify environmental chemical and non-chemical stressors and resiliency factors associated with neurodevelopmental outcomes in childhood and adolescence.

© 2024 Towe-Goodman N et al. *JAMA Network Open.*

			population group were eligible for the current analyses.	
Conditions Affecting Neurocognitive Development and Learning in Early Childhood (CANDLE)	**N=652**	**N=539**	A majority African American sample of mother-child dyads recruited in pregnancy in Memphis, TN from safety net obstetrical clinics and local OB/GYN partners, television and radio advertisements, and directed mailings.	To understand early-life predictors of child socioemotional and neurocognitive development.
The Global Alliance to Prevent Prematurity and Stillbirth (GAPPS)	**N=84**	**N=50**	Pregnant women or women who delivered within 7 days on enrollment from three hospitals in Washington state with racial/ethnic and socioeconomically diverse populations, high pre-term birth rates, and geographic and environmental differences.	To examine how chemical and non-chemical exposures during pregnancy relate to placental gene expression and child neurodevelopmental health and functioning.
Early Parenting of Children (EPoCh)		**N=62**	Sample consists of 200 sibling pairs, recruited from one site in Oregon, in which one sibling was reared from birth with an adoptive family, and the other sibling was reared from birth by the biological mother of the sibling pair. Only children residing with their biological parents were included in the current analyses.	Utilizes a naturalistic human cross-fostering design to advance the understanding of childhood pathways to risk for drug use by identifying nuances in the rearing environment that are associated with child risk behaviors and competencies.
Archive for Research in Child Health (ARCH)	**N=87**		Pregnant women recruited during their first prenatal visit at 1 of 3 prenatal clinics in Lansing, MI.	To examine pre- and perinatal environmental contaminants, nutritional factors, and inflammation in mother-infant dyads in the context of their social and psychological environment on child neurodevelopmental and obesity outcomes.

© 2024 Towe-Goodman N et al. *JAMA Network Open.*

PRogramming of Intergenerational Stress Mechanisms (PRISM)	**N=161**		Pregnant women recruited from prenatal clinics at hospitals and community health centers in Boston, MA and New York, NY, and their offspring.	To examine how prenatal and childhood chemical and non-chemical pro-oxidant environmental factors impact child neurodevelopment, stress regulation, and respiratory health.
ECHO PATHWAYS consortium. The Infant Development and the Environment Study (TIDES)		**N=392**	Pregnant women recruited from prenatal clinics in Minnesota, New York, California, and Washington, and their offspring.	To examine in utero phthalate exposure in relation to sex-specific genital outcomes in infants and neurobehavior in children.

*Cohorts with data from less than 50 participants do not display exact counts

© 2024 Towe-Goodman N et al. *JAMA Network Open.*

eTable 2. Distribution of child, parental, and residential characteristics for early childhood study population by quartiles of NDVI (n = 1,469)*

	**Quartile 1** [0.01-0.27]	**Quartile 2** [0.30-0.33]	**Quartile 3** [0.35-0.38]	**Quartile 4** [0.42-0.57]	**Overall** [0.01-0.57]
	(n=368)	(n=367)	(n=367)	(n=367)	(n=1,469)
Child Characteristics					
Age at CBCL/1½-5, mean (SD)	4.2 (0.6)	4.2 (0.5)	4.2 (0.6)	4.2 (0.6)	4.2 (0.6)
CBCL /1½-5 Internalizing symptoms, t-score, median (IQR)	47 (41, 56)	45 (37, 51)	45 (37, 53)	43 (37, 51)	45 (37, 53)
CBCL /1½-5 Externalizing symptoms, t-score, median (IQR)	46 (39, 52)	44 (39, 50)	44 (39, 52)	44 (37, 51)	44 (39, 51)
Sex assigned at birth					
Male	187 (50.8%)	194 (52.9%)	179 (48.8%)	185 (50.4%)	745 (50.7%)
Female	181 (49.2%)	173 (47.1%)	188 (51.2%)	182 (49.6%)	724 (49.3%)
Child race					
White	180 (49.7%)	189 (51.8%)	164 (45.1%)	164 (44.9%)	697 (47.9%)
Black	93 (25.7%)	122 (33.4%)	159 (43.7%)	154 (42.2%)	528 (36.3%)
Multiple	27 (7.5%)	32 (8.8%)	<40	<45	139 (9.5%)
All others	62 (17.1%)	22 (6.0%)	<10	<5	92 (6.3%)
Child ethnicity					
Non-Hispanic	233 (63.5%)	299 (81.7%)	332 (91.0%)	351 (95.9%)	1215 (83.0%)
Hispanic	134 (36.5%)	67 (18.3%)	33 (9.0%)	15 (4.1%)	249 (17.0%)
Preterm (<37 completed weeks)					
No	339 (92.1%)	330 (89.9%)	342 (93.2%)	343 (93.5%)	1354 (92.2%)
Yes	29 (7.9%)	37 (10.1%)	25 (6.8%)	24 (6.5%)	115 (7.8%)
Birthing Parent Characteristics					
Age at delivery, mean (SD)	28 (6)	29 (6)	28 (6)	29 (6)	29 (6)
Educational attainment					
Less than high school	25 (6.8%)	16 (4.4%)	7 (1.9%)	11 (3.0%)	59 (4.0%)
High school, GED, or equivalent	56 (15.2%)	33 (9.0%)	50 (13.6%)	42 (11.5%)	181 (12.3%)
Some college +	287 (78.0%)	318 (86.6%)	310 (84.5%)	313 (85.5%)	1228 (83.7%)

© 2024 Towe-Goodman N et al. *JAMA Network Open.*

Residential Characteristics					
Neighborhood SES Vulnerability					
High	112 (30.4%)	132 (36.0%)	141 (38.4%)	151 (41.1%)	536 (36.5%)
Medium	88 (23.9%)	97 (26.4%)	78 (21.3%)	76 (20.7%)	339 (23.1%)
Low	168 (45.7%)	138 (37.6%)	148 (40.3%)	140 (38.1%)	594 (40.4%)
Region					
West	215 (58.4%)	152 (41.4%)	37 (10.1%)	9 (2.5%)	413 (28.1%)
Northwest	6 (1.6%)	25 (6.8%)	26 (7.1%)	27 (7.4%)	84 (5.7%)
Midwest/Central	13 (3.5%)	16 (4.4%)	37 (10.1%)	21 (5.7%)	87 (5.9%)
East	134 (36.4%)	174 (47.4%)	267 (72.8%)	310 (84.5%)	885 (60.2%)
Average NDVI @ 270 meters from birth to outcome, mean (SD)	0.21 (0.05)	0.30 (0.02)	0.35 (0.02)	0.42 (0.03)	0.32 (0.08)

* Covariate data are complete or are missing at <1%.

© 2024 Towe-Goodman N et al. *JAMA Network Open.*

eTable 3. Distribution of child, parental, and residential characteristics for middle childhood study population by quartiles of NDVI (n = 1,173)*

	**Quartile 1** [0.03-0.30]	**Quartile 2** [0.30-0.36]	**Quartile 3** [0.36-0.40]	**Quartile 4** [0.40-0.56]	**Overall** [0.03-0.56]
N	(n=294)	(n=293)	(n=293)	(n=293)	(n=1,173)
Child Characteristics					
Age at CBCL/6-18, mean (SD)	7.0 (1.4)	7.7 (1.6)	8.2 (1.6)	8.2 (1.5)	7.8 (1.6)
CBCL/6-18 Internalizing symptoms, t-score, median (IQR)	48 (41, 56)	48 (41, 54)	50 (41, 56)	48 (41, 54)	48 (41, 54)
CBCL/6-18 Externalizing symptoms, t-score, median (IQR)	47 (41, 54)	47 (41, 54)	48 (41, 56)	48 (41, 56)	48 (41, 56)
Sex assigned at birth					
Male	155 (52.7%)	141 (48.1%)	134 (45.7%)	151 (51.5%)	581 (49.5%)
Female	139 (47.3%)	152 (51.9%)	159 (54.3%)	142 (48.5%)	592 (50.5%)
Child race					
White	159 (54.8%)	127 (43.5%)	132 (45.2%)	140 (47.8%)	558 (47.8%)
Black	55 (19.0%)	123 (42.1%)	118 (40.4%)	120 (41.0%)	416 (35.6%)
Multiple	48 (16.6%)	<35	32 (11.0%)	<35	143 (12.3%)
All others	28 (9.7%)	<10	10 (3.4%)	<5	50 (4.3%)
Child ethnicity					
Non-Hispanic	238 (81.0%)	267 (91.4%)	283 (96.6%)	280 (95.6%)	1068 (91.1%)
Hispanic	56 (19.0%)	25 (8.6%)	10 (3.4%)	13 (4.4%)	104 (8.9%)
Preterm (<37 completed weeks)					
No	266 (90.5%)	255 (87.0%)	261 (89.1%)	256 (87.4%)	1038 (88.5%)
Yes	28 (9.5%)	38 (13.0%)	32 (10.9%)	37 (12.6%)	135 (11.5%)
Birthing Parent Characteristics					
Age at delivery, mean (SD)	30 (6)	29 (6)	29 (6)	29 (6)	29 (6)
Educational attainment					
Less than high school	8 (2.7%)	10 (3.4%)	<5	<10	27 (2.3%)
High school, GED, or equivalent	26 (8.9%)	29 (9.9%)	<45	<40	132 (11.3%)

© 2024 Towe-Goodman N et al. *JAMA Network Open.*

Some college +	259 (88.4%)	254 (86.7%)	248 (84.6%)	252 (86.0%)	1013 (86.4%)
Residential Characteristics					
Neighborhood SES Vulnerability					
High	131 (44.6%)	123 (42.0%)	123 (42.0%)	120 (41.0%)	497 (42.4%)
Medium	72 (24.5%)	71 (24.2%)	70 (23.9%)	53 (18.1%)	266 (22.7%)
Low	91 (31.0%)	99 (33.8%)	100 (34.1%)	120 (41.0%)	410 (35.0%)
Region					
West	155 (52.7%)	22 (7.5%)	10 (3.4%)	7 (2.4%)	194 (16.5%)
Northwest	54 (18.4%)	54 (18.4%)	43 (14.7%)	33 (11.3%)	184 (15.7%)
Midwest/Central	24 (8.2%)	47 (16.0%)	33 (11.3%)	13 (4.4%)	117 (10.0%)
East	61 (20.7%)	170 (58.0%)	207 (70.6%)	240 (81.9%)	678 (57.8%)
Average NDVI @ 270 meters from birth to outcome, mean (SD)	0.23 (0.06)	0.33 (0.02)	0.38 (0.01)	0.44 (0.03)	0.35 (0.08)

* Covariate data are complete or are missing at <1%

© 2024 Towe-Goodman N et al. *JAMA Network Open.*

eTable 4. Fully adjusted associations between residential greenspace, individual and area-level confounders, and children's internalizing and externalizing symptoms (CBCL 1½-5/6-18 t-score).

	Early Childhood (2-5 years)		Middle Childhood (6-11 years)	
	Internalizing	*Externalizing*	*Internalizing*	*Externalizing*
Residential				
Lifetime average NDVI (270m)	-1.29 (-1.62, -0.97)	-0.66 (-1.38, 0.06)	0.11 (-0.70, 0.92)	0.20 (-0.51, 0.91)
Neighborhood SES vulnerability* (vs. low vulnerability)				
High SES vulnerability	0.31 (-0.85, 1.48)	1.12 (-0.30, 2.54)	-1.26 (-3.31, 0.80)	1.12 (0.04, 2.20)
Med. SES vulnerability	0.09 (-1.32, 1.50)	0.18 (-0.87, 1.23)	0.13 (-0.84, 1.10)	0.30 (-1.11, 1.71)
Individual				
Birthing-Parent				
Education (vs. <High school)				
High school	-6.27 (-10.07, -2.47)	-5.31 (-8.78, -1.85)	-0.41 (-4.20, 3.38)	-5.59 (-10.94, -0.23)
> High school	-9.45 (-11.96, -6.94)	-6.41 (-8.55, -4.28)	0.93 (-1.79, 3.66)	-4.32 (-9.54, 0.90)
Age at delivery	-0.00 (-0.10, 0.09)	-0.01 (-0.08, 0.06)	-0.05 (-0.13, 0.02)	-0.01 (-0.10, 0.09)
Child				
Female sex at birth (vs. male)	-0.70 (-1.54, 0.13)	-1.98 (-2.70, -1.26)	-1.22 (-2.65, 0.22)	-1.37 (-2.11, -0.63)
Preterm (vs. >37 weeks)	0.95 (0.05, 1.85)	0.34 (-0.46, 1.14)	0.96 (-0.58, 2.51)	1.18 (-0.06, 2.41)

*Neighborhood SES was measured by Social Vulnerability Index (SVI) SES Theme. High social vulnerability corresponds with low SES.

© 2024 Towe-Goodman N et al. *JAMA Network Open.*

eFigure 1: Flowchart for final analytic sample of 2,103 children included in the analysis of residential greenspace and internalizing and externalizing symptoms.

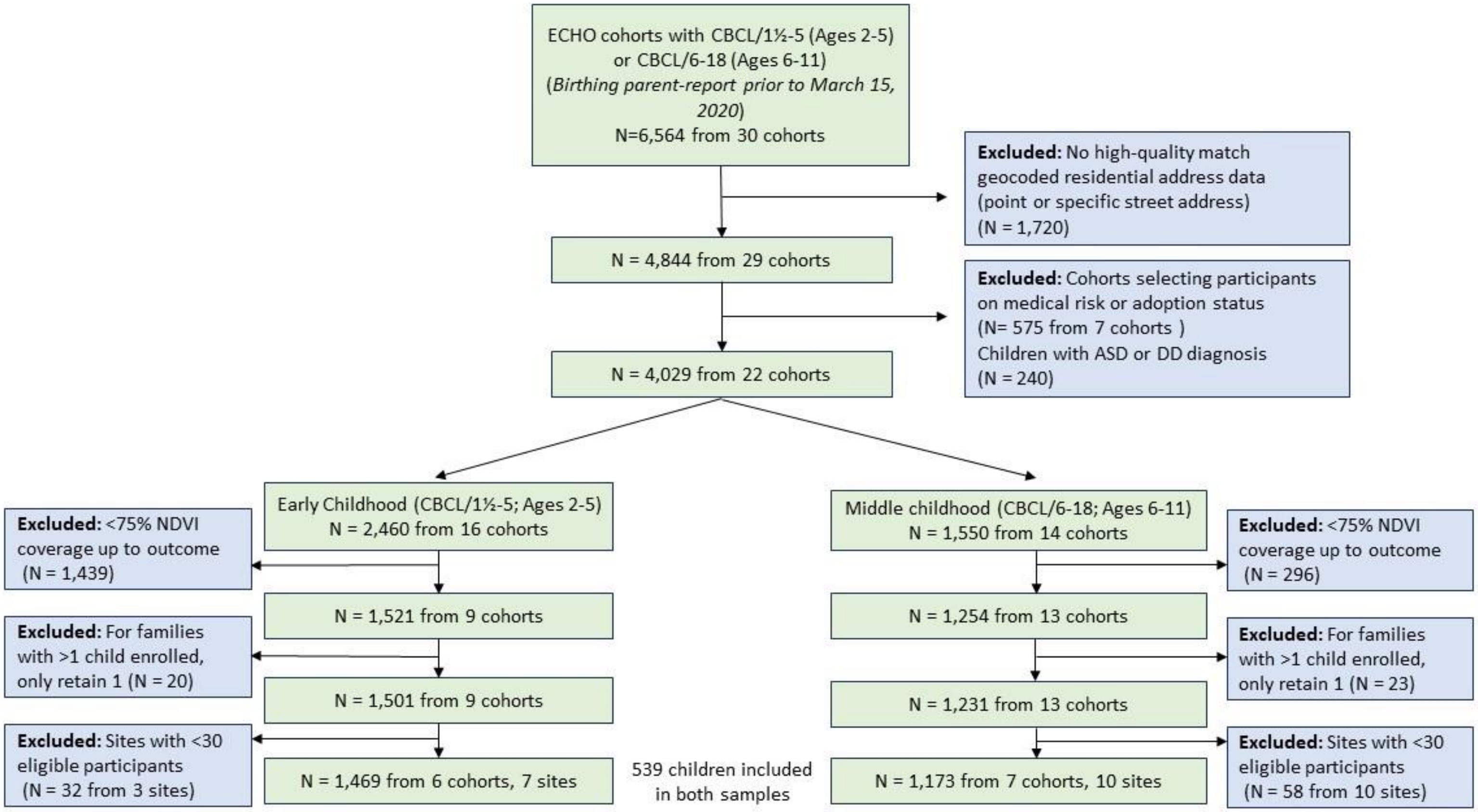

© 2024 Towe-Goodman N et al. *JAMA Network Open.*

eFigure 2: Example counties with average NDVI values at relatively high levels of greenspace (at the 90th percentile of NDVI) for the early childhood sample compared to relatively low levels (the 10th percentile).

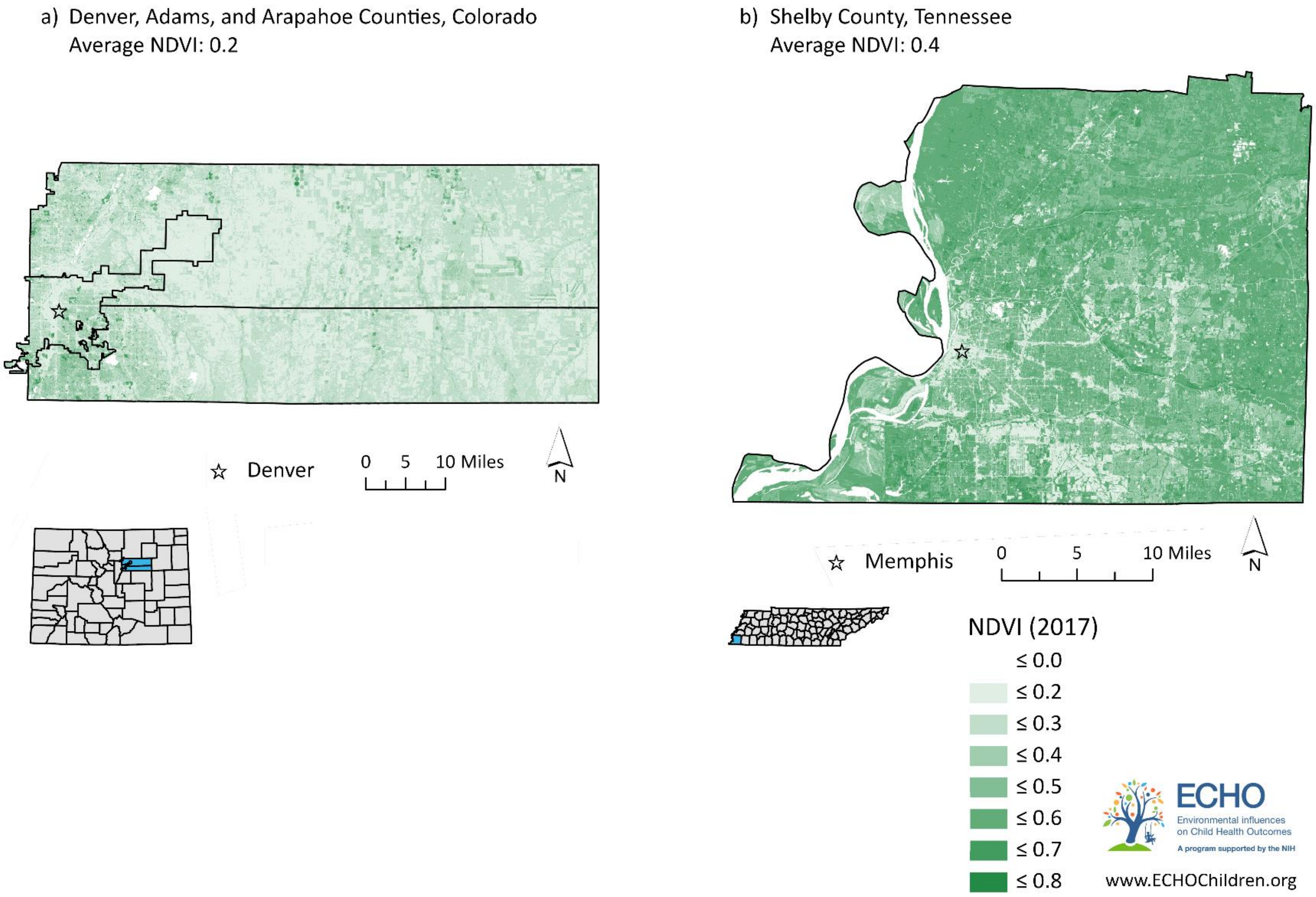

© 2024 Towe-Goodman N et al. *JAMA Network Open.*

eFigure 3: Leave-one-out (cohort enrollment site) coefficient plots for association between greenspace (270m) internalizing and externalizing symptoms for early and middle childhood samples.

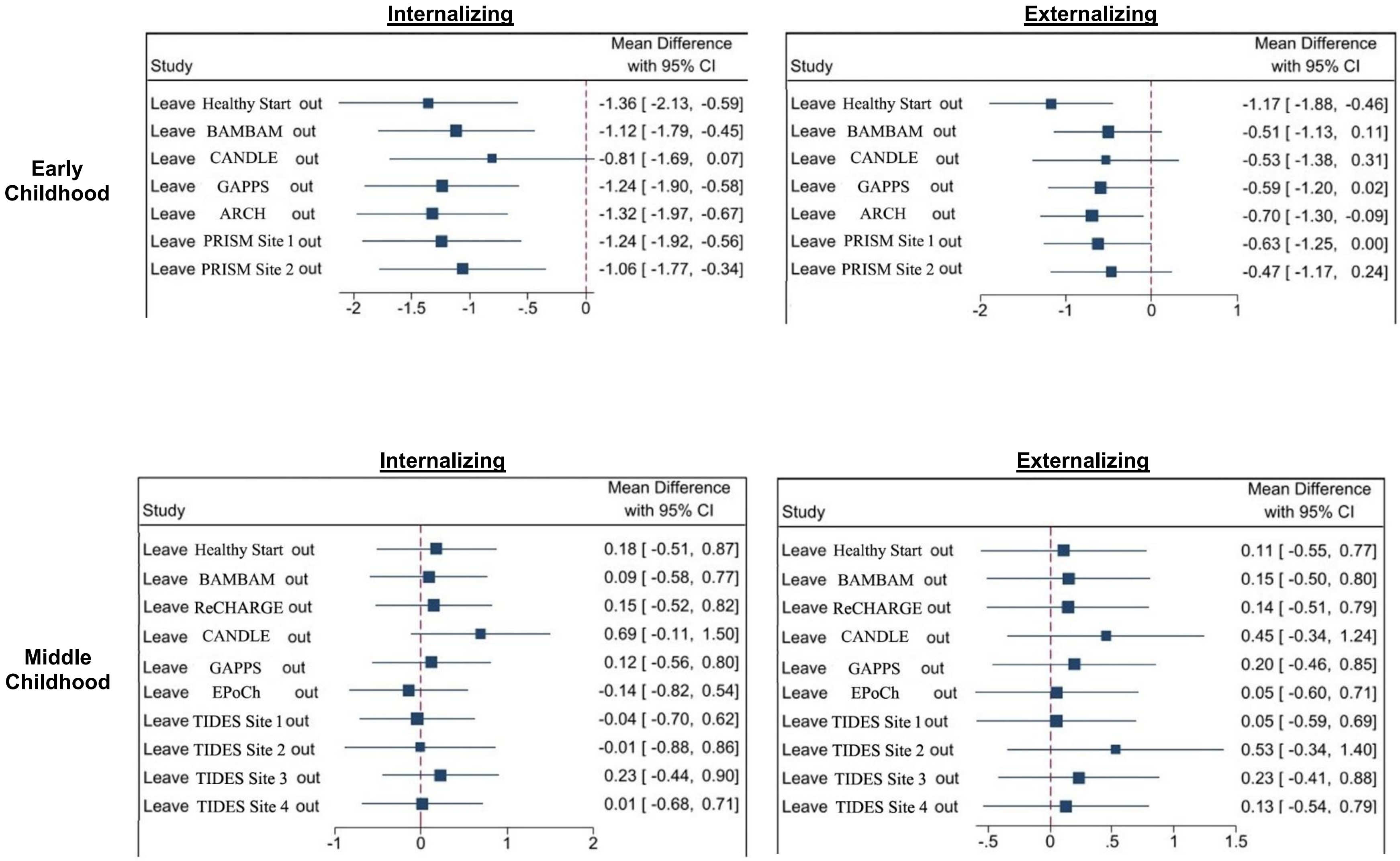

© 2024 Towe-Goodman N et al. *JAMA Network Open.*
