# Supplementary material for: Green Space and Internalizing or Externalizing Symptoms Among Children
Source: JAMA Netw Open. 2024 Apr 10;7(4):e245742. doi: 10.1001/jamanetworkopen.2024.5742 (PMC11007572; doi:10.1001/jamanetworkopen.2024.5742)
Supplement: Supplement 2. — Nonauthor Collaborators [file jamanetwopen-e245742-s002.pdf]

Supplemental Online Content: Nonauthor Collaborators
*First name, last name, and suffix (if applicable) are required and will appear in PubMed.

*Group Name(s): Environmental Influences on Child Health Outcomes program collaborators							
***First Name and Middle Initial(s)**	***Last Name**	***Suffix (eg, Jr, III)**	Academic Degrees	Institution	Location (city, state/province, country)	Role or Contribution, eg, chair, principal investigator	Group (if more than 1 Group listed in the byline) and/or Subgroup (eg, Steering Committee)
P Brian	Smith		MD	Duke Clinical Research Institute	Durham, North Carolina, USA	ECHO Coordinating Center Principal Investigator	ECHO Coordinating Center U2COD023375
L Kristen	Newby		MD	Duke Clinical Research Institute	Durham, North Carolina, USA	ECHO Coordinating Center Prinicipal Investigator	ECHO Coordinating Center U2COD023375
Lisa P	Jacobson		PhD	Johns Hopkins University	Baltimore, Maryland, USA	ECHO Data Analysis Center Principal Investigator	ECHO Data Analysis Center U24D023382
Diane J	Catellier		PhD	Research Triangle Park Institute	Durham, North Carolina, USA	ECHO Data Analysis Center Principal Investigator	ECHO Data Analysis Center U24D023382
Richard C	Gershon		PhD	Northwestern University School of Medicine	Evanston, Illinois, USA	ECHO Person Reported Outcome Core Principal Investigator	ECHO Person Reported Outcome Core U24OD023319
David	Cella		PhD	Northwestern University School of Medicine	Evanston, Illinois, USA	ECHO Person Reported Outcome Core Principal Investigator	ECHO Person Reported Outcome Core U24OD023319
Dana	Dabelea		MD	University of Colorado Denver	Denver, CO, USA	ECHO Cohort Principal Investigator	ECHO Cohort UH3OD023248
Irva	Hertz-Picciotto		MD	University of California Davis Mind Institute	Sacramento, CA, USA	ECHO Cohort Principal Investigator	ECHO Cohort UH3OD023365

Supplemental Online Content: Nonauthor Collaborators
*First name, last name, and suffix (if applicable) are required and will appear in PubMed.

*First Name and Middle Initial(s)	*Last Name	*Suffix (eg, Jr, III)	Academic Degrees	Institution	Location (city, state/province, country)	Role or Contribution, eg, chair, principal investigator	Group (if more than 1 Group listed in the byline) and/or Subgroup (eg, Steering Committee)
Caherine J	Karr		MD	University of Washington, Department of Environmental and Occupational Health Sciences	Seattle, WA, USA	ECHO Cohort Principal Investigator	ECHO Cohort UH3OD023271
Frances	Tylavsky		DrPH, MS	University of Tennessee Health Science Center	Memphis, Tennessee, USA	ECHO Cohort Principal Investigator	ECHO Cohort UH3OD023271
Alex	Mason		PhD	University of Tennessee Health Science Center	Memphis, TN, USA	ECHO Cohort Principal Investigator	ECHO Cohort UH3OD023271
Qi	Zhao		MD, PhD	University of Tennessee Health Science Center	Memphis, Tennessee, USA	ECHO Cohort Principal Investigator	ECHO Cohort UH3OD023271
Sheela	Sathyanarayana		MD	Seattle Children's Research Institute	Seattle, WA	ECHO Cohort Principal Investigator	ECHO Cohort UH3OD023271
Nicole	Bush		PhD	University of California, San Francisco	San Francisco CA, USA	ECHO Cohort Principal Investigator	ECHO Cohort UH3OD023271 and UH3OD023282
Kaja Z	LeWinn		ScD	University of California, San Francisco	San Francisco, California, USA	ECHO Cohort Principal Investigator	ECHO Cohort UH3OD023271
Jody M	Ganiban		PhD	George Washington University	Washington, DC, USA	ECHO Cohort Principal Investigator	ECHO Cohort UH3OD023389
Jenae M	Neiderhiser		PhD	Pennsylvania State University	University Park, PA, USA	ECHO Cohort Principal Investigator	ECHO Cohort UH3OD023389
Jean	Kerver		PhD	Michigan State University	East Lansing, MI, USA	ECHO Cohort Principal Investigator	ECHO Cohort UH3OD023285

Supplemental Online Content: Nonauthor Collaborators
*First name, last name, and suffix (if applicable) are required and will appear in PubMed.

***First Name and Middle Initial(s)**	***Last Name**	***Suffix (eg, Jr, III)**	Academic Degrees	Institution	Location (city, state/province, country)	Role or Contribution, eg, chair, principal investigator	Group (if more than 1 Group listed in the byline) and/or Subgroup (eg, Steering Committee)
Charles	Barone		MD	Henry Ford Health System	Detroit, MI, USA	ECHO Cohort Principal Investigator	ECHO Cohort UH3OD023285
Chris	Fussman		MS	Michigan Department of Health and Human Services (MDHHS)	Lansing, Michigan, USA	ECHO Cohort Principal Investigator	ECHO Cohort UH3OD023285
Nigel	Paneth		MD	Michigan State University	East Lansing, MI, USA	ECHO Cohort Principal Investigator	ECHO Cohort UH3OD023285
Michael R	Elliott		PhD	University of Michigan	Ann Arbor, MI, USA	ECHO Cohort Principal Investigator	ECHO Cohort UH3OD023285
Douglas M.	Ruden		PhD	Wayne State University	Detroit, Michigan, USA	ECHO Cohort Principal Investigator	ECHO Cohort UH3OD023285
Rosalind J	Wright		MD	Icahn School of Medicine at Mount Sinai	New York, NY, USA	ECHO Cohort Principal Investigator	ECHO Cohort UH3OD023337
Michelle	Bosquet-Enlow		phD	Boston Children’s Hospital	Boston MA, USA	ECHO Cohort Principal Investigator	ECHO Cohort UH3OD023337
Leonardo	Trasande		MD	New York School of Medicine	New York, NY, USA	ECHO Cohort Principal Investigator	ECHO Cohort UH3OD023305
Ruby HN	Nguyen		PhD	University of Minnesota	Minneapolis, MN, USA	ECHO Cohort Principal Investigator	ECHO Cohort UH3OD023271 and UH3OD023282
Emily S	Barrett		PhD	University of Rochester Medical Center	Rochester, NY, USA	ECHO Cohort Principal Investigator	ECHO Cohort UH3OD023271 and UH3OD023282

Supplemental Online Content: Nonauthor Collaborators
*First name, last name, and suffix (if applicable) are required and will appear in PubMed.

***First Name and Middle Initial(s)**	***Last Name**	***Suffix (eg, Jr, III)**	Academic Degrees	Institution	Location (city, state/province, country)	Role or Contribution, eg, chair, principal investigator	Group (if more than 1 Group listed in the byline) and/or Subgroup (eg, Steering Committee)
Emily	Oken		MD, MPH	Harvard Medical School	Boston, MA, USA	ECHO Cohort Principal Investigator	ECHO Cohort UH3OD023286
Robert O	Wright		MD, MPH	Icahn School of Medicine at Mount Sinai	New York, NY, USA	ECHO Cohort Principal Investigator	ECHO Cohort UG3/UH3OD023337
Sean CL	Deoni		PhD	Brown University	Providence, RI, USA	ECHO Cohort Principal Investigator	ECHO Cohort UG/UH3OD023313
Daphne M	Koinis-Mitchell		PhD	Brown University	Providence, RI, USA	ECHO Cohort Principal Investigator	ECHO Cohort UG/UH3OD023313
Manish	Arora		BDS, MPH, PhD	Icahn School of Medicine at Mount Sinai	New York, NY, USA	NIEHS Principal Investigator	NIEHS P30ES023515
Lianne (Elizabeth A)	Sheppard		PhD	University of Washington	Seatle, WA, USA	NIEHS Principal Investigator	NIEHS T32ES015459
Shanna H	Swan		PhD	Icahn School of Medicine at Mount Sinai	New York, NY, USA	ECHO Cohort Principal Investigator	ECHO Cohort UH3OD023271
James	Cajka		MA, BSc	RTI International	Research Triangle Park, NC	Processing NDVI Data	ECHO Data Analysis Center U24D023382
Clancy	Blair		PhD	New York School of Medicine	New York, NY, USA	ECHO Cohort Principal Investigator	ECHO Cohort UH3OD023332
W. Benjamin	Goodman		PhD	Duke University	Durham, North Carolina, USA	Manuscript Review	
