# Supplementary material for: Green Space and Internalizing or Externalizing Symptoms Among Children
Source: JAMA Netw Open. 2024 Apr 10;7(4):e245742. doi: 10.1001/jamanetworkopen.2024.5742 (PMC11007572; doi:10.1001/jamanetworkopen.2024.5742)
Supplement: Supplement 3. — Data Sharing Statement [file jamanetwopen-e245742-s003.pdf]

Data Sharing Statement

Towe-Goodman. Greenspace and Internalizing or Externalizing Symptoms Among Children. *JAMA Netw Open*. Published April 10, 2024. doi:10.1001/jamanetworkopen.2024.5742

Data
Data available: Yes
Data types: Deidentified participant data
How to access data: https://dash.nichd.nih.gov/
When available: With publication

Supporting Documents
Document types: None

Additional Information
Who can access the data: Researchers whose proposed use of the data has been approved
Types of analyses: By accepting the NICHD DASH User Agreement, you agree: to use NICHD DASH for the purposes of archiving and accessing data obtained from scientific research with the intent of data reuse to use NICHD DASH data for scientific research in an institution with an approved assurance from the Department of Health and Human Services Office for Human Research Protections, and to not use the data for commercial purposes (or sell the data obtained from NICHD DASH) to preserve and protect the confidentiality of, and not attempt to identify, any individuals or households in the data that archived data in NICHD DASH are provided without warranty or liability of any kind to notify the NICHD DASH Administrator of any errors discovered in the archived data to establish safeguards to prevent unauthorized viewing or release of NICHD DASH information or data to comply with any charges that may apply for various services offered by NICHD DASH to ensure that the means of access to NICHD DASH (such as passwords) are kept secure and not disclosed to anyone else that personal data submitted by you are accurate to the best of your knowledge and kept up to date by you that personal data provided by you may be used for administrative management of NICHD DASH and for reporting purposes with the goal of improving services offered by NICHD DASH that any breach of the NICHD DASH User Agreement could lead to termination of your access to the services
Mechanisms of data availability: With approved NICHD DASH User Agreement
Any additional restrictions: Users of DASH agree to comply with all terms and conditions of the NICHD DASH User Agreement during the registration process.
